# Knowledge, attitude and practices of malaria preventive measures among mothers with children under five years in a rural setting of Ghana

**DOI:** 10.1186/s12936-023-04702-3

**Published:** 2023-09-12

**Authors:** Prince Adum, Veronica Adwoa Agyare, Joseph Owusu-Marfo, Yaa Nyarko Agyeman

**Affiliations:** 1 Kintampo Municipal Government Hospital, Bono East Region, Kintampo, Ghana; 2 Ghana College of Nurses and Midwives, Kumasi, Ashanti Region Ghana; 3 Department of Epidemiology, Biostatistics and Disease Control, School of Public Health, University for Development Studies, Tamale, Northern Region Ghana; 4 Department of Population and Reproductive Health, School of Public Health, University for Development Studies, Tamale, Northern Region Ghana; 5 Ministry of Health Training Institution, SDA Nursing & Midwifery Training College Kwadaso, Kumasi, Ghana

**Keywords:** Attitude, Children under 5 years, Factors, Knowledge, Malaria, Mothers, Practice, Prevention

## Abstract

**Background:**

Malaria remains a major public health concern around the world, particularly in resource-constrained countries. Malaria still accounts for 40% of all Out-Patient Department (OPD) cases in Ghana, with children under the age of five being the most vulnerable group. The study assessed the knowledge, attitudes, and practices of malaria preventive measures among mothers with children under 5 years old in a rural setting in Ghana.

**Methods:**

A cross-sectional study design with a quantitative approach was used in this study. The study was facility based and involved the use of interviewer administered questionnaires to collect data from 281 mothers with children under the age of five. Simple random sampling method was used to select the respondents. The data collected was analysed using the statistical package for the social sciences (SPSS) version 22 and results presented in tables.

**Results:**

There were 281 mothers, with 59.4% having children at the age of a year. The findings revealed that the majority of participants have a high level of knowledge about malaria’s causes, signs, and symptoms. Again, the majority of participants demonstrated a positive attitude toward malaria prevention, such as seeking treatment at a hospital within 24 h of suspecting their children had malaria and demonstrating good knowledge of malaria prevention practices. Despite this, 35.5% of respondents were not actively engaged in malaria prevention practices in a day prior to the interview. Respondents’ occupation, level of education, and religion had a statistically significant association with mothers’ attitude towards prevention (p-values < 0.05 and 0.01).

**Conclusion:**

The study’s findings clearly demonstrate that the majority of mothers were knowledgeable about the causes, signs and symptoms, and preventive measures of malaria in children under the age of five. There was also statistically significant association between mothers’ demographic information, including level of education, occupation, religion, and their attitude towards malaria prevention. A keen interest should be directed toward the consistent application of low-cost preventive measures.

## Background

Despite significant efforts over the century to eradicate or manage malaria, it continues to be a serious public health problem throughout the world, affecting more pregnant women and children due to their vulnerability [1]. Malaria has been regarded as a long-standing global health problem because of the high morbidity and death rates among pregnant women and children [2]. In addition to that, malaria is viewed as a life-threatening condition caused by parasites that are transferred to people by the bites of infected female Anopheles mosquitoes [3]. Malaria is one of the most deadliest infectious disease, with an estimated 229 million cases and 409,000 deaths worldwide in 2019 [4]. According to Limoukou et al. [4], children under the age of five accounted for 67% (274,000) of all malaria fatalities worldwide. The African Region of the World Health Organization (WHO) bears a disproportionately large share of the global malaria burden, accounting for 94% of malaria cases and fatalities in 2019 [5]. In addition, malaria was responsible for more than 18% of under-five deaths in sub-Saharan Africa in 2019 [6].

Malaria is noted to be more geographically distributed in regions with warm climates and high levels of mosquito activity [7]. Therefore, extensive public health promotion initiatives are required in Africa to achieve long-term malaria control by focusing on contemporary and proven malaria prevention and management strategies, as well as the need to investigate the importance of malaria transmission with an emphasis on knowledge, attitude, and practices (KAP) [8]. Malaria’s poor outcomes were attributed to ineffective treatment and inadequate maternal knowledge of preventive measures, examples; poor logistics systems leading to undersupply of drugs and diagnostics, health workers issues, lack of self efficacy of women to provide the treatment as required, socio-cultural determinants of health, side effects management, partial drug resistance [9].

Ghana has not been left out, as it continues to battle malaria cases in children under-fives. According to Anaba et al. [10], Ghana recorded 2.3 million cases of malaria in out-patient department (OPD) in the first quarter of 2017. This figure represents a 1.5% increase over the number of cases reported in the first quarter of 2016 [10]. A nationwide survey in Ghana found that the average parasite prevalence among children aged 6 to 59 months was 28%. [11]. Malaria is also responsible for 40% of all out-patient visits in Ghana, with children under the age of five and pregnant women being the most vulnerable groups [12]. Over the last 2 years, significant efforts have been made to strengthen malaria prevention, as it remains the leading cause of death in Ghana, primarily among children under the age of five [13]. This demonstrates that the government’s efforts to strengthen malaria prevention are still insufficient to reduce the death rate. According to the evidence, strategies for controlling malaria in an endemic country include health education, changing attitudes and practices [14]. KAP studies are also important for developing epidemiological and behavioural baselines in malaria surveillance programmes, because misconceptions about malaria, for example, can lead to self-medication and incorrect use of insecticide-treated nets. As a result, assessing the population’s KAP in relation to malaria prevention is critical [15]. Despite some success in lowering malaria morbidity and mortality rates, some rural districts, such as Kintampo North, continue to have a high prevalence of malaria cases when compared to urban areas. According to Kwofie et al. [16], the rural and urban areas have 28% and 4% of the population, respectively. Adequate malaria knowledge among mothers has a strong correlation with lower morbidity and mortality among children under the age of five [17]. The investigation of local communities’ malaria KAP in Ghana is critical for the design and implementation of effective malaria control programmes [18]. Malaria cases increased marginally (1659, 1737, 1983) from 2016 to 2018, then decreased (1392) in 2019, with a further decrease to (1023) in 2020, according to Kintampo North Municipal Hospital annual reviews. Though there has been some progress, the numbers remain in the thousands and have not decreased significantly. The decrease in 2020 could be attributed to underreporting to hospitals as a result of the covid-19 pandemic. Several malaria studies have been conducted in Kintampo North Municipality, according to the literature reviewed [19–25]. Of all these studies conducted in Ghana, only the study by Anim [25] examined factors associated with reported severe malaria among children under 5 years old at the Kintampo Municipal Hospital. However, it did not specifically focus on prevention strategies. It has therefore become very clear that, to curtail the increasing cases of malaria in the municipality, much attention is required on the prevention of the malaria infection. The study aimed at assessing the KAP of malaria preventive measures among mothers with children under 5 years using a cross sectional study design. Only Anim [25] looked at the factors associated with reported severe malaria among children under 5 years old in the Kintampo Municipal Hospital, and even then, it was not focused on prevention. As a result, it is clear that, in order to reduce the rising number of malaria cases in the municipality, much emphasis must be placed on malaria infection prevention. A cross-sectional study design was used to assess the KAP of malaria preventive measures among mothers with children under 5 years old.

## Methods

### Study setting

The research was carried out at Kintampo North Municipal Hospital. Kintampo North Municipality, with Kintampo as its capital, was established in 1988 by legislative instrument 1480 as part of the Government’s decentralization programme. Kintampo North Municipality is one of 11 districts that comprise the newly formed Bono East Region. The total land area occupied is approximately 5108 km^2^ (1192 sqmi). The total population is 109,448 people, with 4337 pregnant women and 21,889 children under the age of five. The municipality is strategically located in the centre of Ghana, serving as a transit point between the country’s northern and southern sectors. It is located between latitudes 80 45’ N and 70 45’ N, and longitudes 10 20’ W and 20 1’ E. The West African Frontier Force established the Kintampo Municipal Hospital as a clinic in 1940, and it was later upgraded to a district hospital. The Kintampo Health Research Centre and the College of Health and Wellbeing share the hospital’s boundaries and are located in the township’s heart. The research facility is the only referral facility in Kintampo North Municipality at the moment. The hospital has 160 beds, 3 medical officers, 10 physician assistants, and 200 nurses. The hospital provides medical, surgical, radiological, and laboratory services. The OPD and the Paediatric ward served as research recruitment points. The Paediatric ward can accommodate 30 patients.

### Study design and sample size

A cross-sectional study design was employed with a quantitative approach. The study engaged mothers with children under 5 years old within the Kintampo north Municipal in the Bono East Region of Ghana. In all, a total of 281 mothers were selected for the study. Taro Yamane method of calculating sample size was used to determine the number of respondents needed for this study [26]:

n = N/(1 + N(e)^2^)

where n signifies the sample size, N signifies the population understudy, e signifies the margin of error (0.05), n = 1,000/ (1 + 1,000(0.05)^2^), n = 1,000/3.6, n = 277.77, n = 278.

An assumed non-response rate of 1% was calculated on the sample size of 278. Thus, the final sample size of 281 of mothers with children under 5 years were recruited for this study.

### Sampling technique

The study employed a facility-based sampling method, specifically targeting participants within the chosen healthcare facility. Within this facility, a combination of sampling techniques was used to ensure a diverse representation of mothers with children under the age of five. These techniques included selecting mothers who visited the emergency department, outpatient department (OPD), mothers of children admitted to the paediatric ward, and those who were able to communicate verbally. To minimize any potential bias in participant selection, a table of random numbers was utilized. However, it’s important to note that the choice of the study facility itself was intentional, as it served as the primary treatment centre catering to children under the age of five in the surrounding area. All mothers who met the inclusion criteria and were willing to participate were considered eligible for the study. It is worth mentioning that informed consent, obtained through signing or thumbprinting an informed consent form, was a prerequisite for participation. Mothers who did not provide such consent were excluded from the study. Participation was completely voluntary for all the respondents in the study facility.

### Data collection instruments and procedure

The primary data collection instrument in this study was a structured questionnaire based on questions from similar published articles [4, 15, 27]. This study’s questionnaire included an introduction section with consent to participate, as well as four sections: Section A was the socio-demographic data, which included mothers’ age, marital status, number of children under the age of five, religion, educational level, and occupation. Section B dealt with mothers’ malaria knowledge, Section C with mothers’ malaria prevention practices, and Section D with mothers’ attitudes toward malaria prevention. Data was collected over a four-week period, from July 12th, 2021 to August 11th, 2021. The questionnaire was created in an electronic form on a computer using the Google Forms web application to minimize contact with study participants in order to control the spread of COVID-19 infection as well as the quality of data collected. The research assistants were trained on how to use Google forms to administer the questionnaire to the respondents one at a time. The link to the Google form was shared with those who could read and understand. However, for those who were unable to read or write, research assistants assisted them in answering electronic questionnaires by reading to them and selecting the appropriate response of the participants. The electronic questionnaire was read to the participants and translated in their preferred local language(s).

The following procedures were taken to ensure uniformity in the translation of the electronic questionnaire for participants who desired it to be read to them. First, qualified bilingual individuals were chosen who were fluent in both the questionnaire language and the participants’ preferred local language. These people were educated on the study procedure, questionnaire content, and the necessity of consistency in translation. These trained professionals read the questions aloud to the participants in their preferred local language throughout the administration of the questionnaire. All participants were treated with care to guarantee proper translation and consistency in tone, emphasis, and clarity. Any concerns or explanations requested by participants were consistently answered, in accordance with pre-established guidelines supplied to trained individuals. Several procedures were adopted to avoid bias in order to examine the potential discrepancies in replies between self-administered questionnaires and research assistant interviews. Interview procedures were thoroughly taught to research assistants, including the significance of neutrality, non-directiveness, and avoiding leading questions. They were told not to give further explanations or emphasize specific sections of the questions unless specifically requested by the participant. Furthermore, the interviewing skills of research assistants were regularly monitored and supervised to guarantee conformity to the specified guidelines. Any deviations or biases discovered throughout the monitoring phase were addressed immediately by retraining and reinforcing interview procedures. Although steps were made to reduce bias, it is vital to recognize that the presence of research assistants may have introduced some level of bias, such as accidental influence or urging. Attempts were made, however, to minimize these possible biases and maintain consistency in data collection across both self-administered questionnaires and research assistant interviews by giving explicit rules and regular supervision.

#### Pre-testing

The questionnaire was pre-tested on 20 randomly chosen mothers with children under 5 years old at the Sunkwa Clinic (a private rural clinic located in the Kintampo Municipality), and their feedback was used to update the questionnaire before the actual data collection began on the 11th of July, 2021. This was also to make the research assistants conversant with the electronic questionnaire.

#### Data processing and analysis

The data collected with the Google forms were exported to excel, validated, cleaned before importing into the IBM statistical package for the social sciences (SPSS) version 22 for data analysis. The data were analysed based on the study objectives and the main study variables. Descriptive statistics (frequencies and their percentages) and were computed and summarized in simple tables for categorical variables. Partial correlation of demographic variables and mothers’ attitude towards malaria prevention was also tested. All the results were summarised and presented in tables. Class intervals were also computed for percentages based on the study’s confidence interval of 95%.

## Results

Most of the mothers were between the ages of 30 to 34 years old (35.2%), married (76.9%) and had one child under the age of five (59.4%). The median age among the mothers was 30–34 years group. Majority of the respondents were Christians (50.5%), while most of them had tertiary level of education (28.5%). The least category of mother’s education level was primary education representing 14.2%. The majority of the respondents were self-employed (35.6) with civil servants forming the least (9.6%) (Table 1).

**Table 1:** Demographic characteristics of respondents

Variables	Frequency n (%)
*Age group of mothers*	
15–19	10 (3.6)
20–24	33 (11.7)
25−24	82 (29.2)
30–34	99 (35.2)
35 and above Median age 30–34	57 (20.3)
*Marital status*	
Married	216 (76.9)
Widowed	6 (2.1)
Divorced	15 (5.3)
Single	44 (15.7)
*No. of children* *under 5 years*	
1	167 (59.4)
2	92 (32.7)
3 or more	22 (7.8)
*Religion*	
Christianity	142 (50.5)
Islam	130 (46.3)
Traditional	7 (2.5)
Other	2 (0.7)
*Educational level*	
Primary	40 (14.2)
JHS/middle	54 (19.2)
Secondary	51 (18.1)
Tertiary	80 (28.5)
No formal education	56 (19.9)
*Occupation*	
Farming	76 (27.0)
Self employed	100 (35.6)
Civil servant	27 (9.6)
Other	78 (27.8)

Most of the mothers interviewed had heard about malaria before (99.6%) (95% Cl: 98.9, 100). The dichotomous variable, with only two possible answers, was used for the question about whether physical contact can transmit malaria. In this case, 9.3% of them answered Yes (95% Cl: 5.7, 13.5), while 90.7% (Table 2).

**Table 2:** Mothers knowledge level on malaria

Variables	Frequency n (%)	95% Cl (lower, upper)
*Have you heard about malaria*		
Yes	280 (99.6)	(98.9, 100)
No	1 (0.4)	
*Does physical contact transmit malaria*		
Yes	26 (9.3)	(5.7, 13.5)
No	255 (90.7)	

Table 3 presents the main causes of malaria, including too much sun, a dirty environment, witchcraft, and mosquitoes. In all, 49.5% of the participants indicated mosquitoes as a factor for causing malaria. Meanwhile, mosquitoes were implicated in 97.5% of the multiple factors that cause malaria. The results in Table 4 listed the signs and symptoms of malaria as weakness, fever, and loss of appetite. Also, the study listed ‘all of the above (all)’ and ‘none of the above’ as possible answers. Majority (52.7%) of the respondents selected ‘all’ (weakness, fever and loss of appetite) as the signs and symptoms of malaria.

**Table 3:** Mother’s level of knowledge on malaria (continuous)

Variables	Frequency n (%)
*Causes of malaria*	
Too much sun only	1 (0.4)
Too much sun, dirty environment and mosquitoes	15 (5.3)
Too much sun and mosquitoes	11 (3.9)
Too much sun and dirty environment	2 (0.7)
Dirty environment only	1 (0.4)
Dirty environment and mosquitoes	103 (36.7)
Witchcraft only	1 (0.4)
Mosquitoes only	140 (49.8)
Don’t know only	7 (2.5)

**Table 4:** Mother’s level of knowledge on malaria

Respondence responses	Frequency n (%)
*Signs and symptoms of malaria*	
Weakness only	6 (2.1)
Weakness and fever	20 (7.1)
Weakness, fever and loss of appetite	12 (4.3)
Weakness, fever, loss of appetite and all	38 (13.5)
Weakness, fever and all	1 (0.4)
Weakness and loss of appetite	2 (0.7)
Weakness, fever and all	1 (0.4)
Fever only	12 (4.3)
Fever and loss of appetite	17 (6.0)
Fever, loss of appetite and all	6 (2.1)
Fever and all	5 (1.8)
All	148 (52.7)
Loss of appetite only	1 (0.4)
Loss of appetite and all	1 (0.4)
Don’t know	11 (3.9)

All: represents those who were able to select all the signs and symptoms

The results in Table 5 show mothers’ attitudes towards malaria prevention, including whether it is possible to recover from malaria without treatment, whether anyone can become infected with malaria, whether they seek health care when their children exhibit signs and symptoms of malaria, how quickly mothers seek treatment when their children are sick, and where they seek that treatment. With the possibility of recovering from malaria without treatment, the options were Yes or No. Most of the respondents (81.1%) responded No. It was discovered that 254 out of a total of 281 participants (90.4%) seek treatment at a hospital. It was also revealed that 5.3% use drugs at home and 4.3% use herbal preparations. When asked if anyone could contract malaria, 93.2% said yes. When it comes to how quickly respondents seek treatment, 46.3% seek treatment within 12 h, 42.7% within 24 h, 7.8% within 48 h, and 1.4% within 72 h. Most of the mothers know that malaria is a life-threatening disease (96.1%), seek medical attention when their children show signs and symptoms of malaria (98.2%) and dangerous not to complete malaria treatment (89.7%).

**Table 5:** Mothers attitude towards malaria prevention

Variables	Frequency n (%)
*Do you seek healthcare*	
Yes	276 (98.2)
No	5 (1.8)
*How prompt do you seek treatment*	
Within 12 h	130 (46.3)
Up to 24 h	120 (42.7)
Up to 48 h	22 (7.8)
Up to 72 h	9 (3.2)
*Where do you seek treatment*	
Go to hospital	254 (90.4)
Take drugs at home	15 (5.3)
Give herbal preparation	12 (4.3)
*Malaria is a life-threatening disease*	
Yes	270 (96.1)
No	11 (3.9)
*It is dangerous not to complete malaria treatment*	
Yes	252 (89.7)
No	29 (10,3)
*Anyone can get malaria*	
Yes	262 (93.2)
No	19 (6.8)
*Possible to recover from malaria without treatment*	
Yes	53 (18.9)
No	228 (81.1)

Table 6 depicts the association between the demographic information of the respondents, such as; (age, marital status, number of children under 5 years, religion, level of education and occupation) and the attitude of the mothers towards malaria prevention. Respondents’ occupation had a statistically significant association with mothers’ attitude towards malaria prevention (p-values < 0.05 and 0.01). Also, mothers’ marital status had a statistically significant association with mothers’ attitude towards malaria prevention such as; Malaria being a life-threatening disease (p-value < 0.05), promptness of seeking malaria treatment (p-value < 0.01), where to seek treatment (p-value < 0.01) and the danger not completing malaria treatment (p-value < 0.01). Other demographic information, such as religion, educational level and mothers’ number of children under 5 years of age had statistically significant association with mothers’ attitude towards malaria prevention.

**Table 6:** Partial correlation of demographic variables and mothers’ attitude towards malaria prevention

Measurement	1	2	3	4	5	6	7	8	9	10	11	12	13
Age of mother (1)	1.00												
Marital status (2)	− 0.231^**^	1.00											
Number of children under 5 years (3)	0.281^**^	− 0.141^*^	1.00										
Religion (4)	0.130^*^	0.039	0.079	1.00									
Mother’s level of education (5)	0.152^*^	0.021	0.077	− 0.058	1.00								
Occupation (6)	− 0.194^**^	0.033	− 0.154^**^	− 0.238^**^	0.189^**^	1.00							
Malaria health care seeking when child sick (7)	0.057	− 0.005	0.089	0.100	− 0.046	− 0.147^*^	1.00						
How prompt do seek treatment (8)	0.097	0.162^**^	0.125^*^	0.090	− 0.023	− 0.332^**^		1.00					
Where do You seek treatment (9)	− 0.113	0.189^**^	0.029	0.102	0.084	− 0.264^**^	0.236^**^	0.424^**^	1.00				
Malaria is a life-threatening disease (10)	0.081	0.135^*^	0.102	0.155^**^	0.174^**^	− 0.141^*^	0.250^**^	0.242^**^	0.375^**^	1.00			
Dangerous not to complete malaria treatment (11)	− 0.033	0.173^**^	0.067	0.117	0.173^**^	− 0.285^**^	0.220^**^	0.400^**^	0.645^**^	0.535^**^	1.00		
Anyone can get malaria (12)	0.025	0.081	0.054	0.144^*^	0.068	− 0.169^**^	0.285^**^	0.266^**^	0.450^**^	0.457^**^	0.514^**^	1.00	
Possible to recover from malaria (13)	− 0.047	− 0.008	− 0.102	0.044	0.038	0.227^**^	− 0.142^*^	− 0.129^*^	− 0.153^*^	− 0.184^**^	− 0.165^**^	− 0.124^*^	1.00

**Correlation is significant at the 0.01 level (2-tailed)

*Correlation is significant at the 0.05 level (2-tailed)

## Discussion

The socio-demographic characteristics of the study is relevant in the prevention of malaria. Individuals’ educational attainment and their age emerged as particularly important factors for understanding the etiology of malaria. With education, the results revealed that all individuals with at least primary level of education correctly attributed the cause of malaria to a mosquito. This was in line with a study conducted in Douala, Cameroon [28], which found that the level of education was associated with the correct knowledge on the causative agent of malaria. It is, therefore, not surprising of the findings as governmental interventions to increase access to education in Ghana could have an additional effect of improving the overall health outcomes of the under-five population regarding malaria prevention in future. The relevance of age was also a factor, with the majority of respondents being between the ages of 20 and 35. It should be noted, however, that age alone does not determine an individual’s awareness or capacity in safeguarding their children from malaria. Education, access to information, and awareness campaigns may have a greater impact on knowledge levels about malaria prevention and child protection [15].

The current study revealed that majority of the participants knew that mosquitoes were the main causes of malaria among children despite the fact that they also added some other causes such as too much sun, witch craft, dirty environment, busy area. This is consistent with a study conducted by Asuamah et al. [29], in Northern Ghana where majority of the students knew about the cause of malaria. Also, a study among pregnant women in Kassena-Nankana East showed that overwhelming majority of study participants knew that mosquitoes causes malaria [30]. Similarly, the study by Talipouo et al. [1] in the city of Yaoundé noted that a majority of the women believed malaria was caused by mosquito bite. According to the WHO Global report [5], Ghana is among the 15 countries globally with the highest burden of malaria. Ghana is among the many countries that have adopted malaria prevention campaigns to reduce the incidence of malaria in the continent especially among pregnant women and children under 5 years. These campaigns adopted by the Movement of Ghana (MOG) could explain the higher appreciation of the cause of malaria. Similarly, there are deliberate attempts by the Ministry of Health through its agencies with the support of Non-Governmental Organizations, such as WHO or UNICEF, to increase knowledge on malaria prevention. These attempts in Ghana could be the reason for higher study participants knowing the actual cause of malaria. Due to the support received by most African countries on malaria prevention, it is difficult to get contrary views on the cause of malaria [31, 32]. The high level of knowledge realized in this current study about signs and symptoms of malaria, agrees with a study conducted in South African on the knowledge, attitude and practices on malaria transmission at the Mamfene and KwaZulu—Natal Province where majority of the participants were able to identify three or four signs or symptoms of malaria [33]. This trend was also observed in Cameroun, where almost all the participants (90%) recognized the signs and symptoms of malaria [15]. This could be due to the fact that they are higher proportions of participants being educated and mature in age. This current study also had (99.6%) of participants who had heard about malaria. This finding corroborated with some studies in Ghana [34–36] where majority of the respondents have heard about malaria. Majority of the participants in this current study also attributed their source of malaria information to radio and television. These findings are similar to those of Asumah et al. [30] where the media was the major source of information on malaria issues. In the Kintampo Municipality in the Bono East of Ghana, the public health unit visits the radio stations twice every week to educate the population on disease conditions and their preventions. It could be that with the radio talk shows, these women are comfortable in their homes and listen attentively. Also, they can call into the programme to seek clarity. This could explain why majority of the current study participants obtained information on malaria via the media (Radio and TV). However, it should be noted that individual preferences and circumstances may influence the level of engagement. Factors such as the timing of the radio messages, including potential conflicts with meal preparation or water fetching time, could affect the listenership and attentiveness of women. This study revealed that majority of mothers have good attitude towards malaria prevention by seeking healthcare within 24 h when child experiences signs and symptoms of malaria in spite of some few misconceptions. This is in contradiction with another study conducted in Ghana at Asutsuare, a rural irrigated farming community in the Greater Accra Region which revealed that only 3% immediately visit a health facility for treatment whenever they suspected they had malaria [18]. The overwhelming majority indicated they only visit a healthcare facility for treatment if they felt the suspected malaria illness was severe or other treatment options had failed. The difference in the above studies could be due to the fact that this study only used mothers and was also conducted in the hospital whereas [9] study was conducted among a whole community which have different people from different ethnic groups and that could also have influenced the late reporting. Similar studies conducted in different countries in Africa [9, 37, 38] also affirms to the findings of this study on the conclusion that, despite some respondents delay in seeking health care for their children under 5 years, majority seek health care within 12 to 24 h when they suspected malaria infection. This is a good sign to improve child under five mortalities. Respondents’ occupation had a statistically significant association with mothers’ attitude towards malaria prevention. Also, mothers’ marital status had a statistically significant association with mothers’ attitude towards malaria prevention such as; Malaria being life-threatening disease, promptness of seeking malaria treatment, where to seek treatment and the danger not completing malaria treatment. Other demographic information such as; religion, educational level and mothers with number of children under 5 years of age had statistically significant association with mothers’ attitude towards malaria prevention. These studies have shown that education about attitude towards seeking healthcare at the right time should be intensified to save all children under 5 years.

Shockingly, 18.9% of the mothers in this study believe they could recover from malaria without taking any treatment. Malaria ought to be treated at all cost to avoid the cascading complication, such as anaemia [39]. The sickness pose by malaria can also make the mother weak hence her inability to breastfeed on demand. This suggest that for mother to think they could actually recover from malaria without taking medications; they tend to risk the safety of their babies [40]. Therefore, if the right drugs are used, people who have malaria can be cured and all the malaria parasites can be cleared from their body. However, the disease can continue if it is not treated or if it is treated with the wrong drug. The motivation for this is not known as far as the researcher is concern. The study, therefore, recommends more in-depth research to unravel the thinking that goes into this choice by the mothers.

In relation to above, the study further established that majority of the participants felts its dangerous not to complete the malaria treatment. Malaria parasite-based diagnosis is uncommon in the private health sector, particularly in the informal private retail sector dominated by patent and proprietary drug suppliers (PPMVs). There is availability of malaria test kits in every pharmacy. Just like any other laboratory test, they are false positives and false negative. Some do not even test to know their status [41]. As a result, malaria drugs are sold and used without parasitological proof. This overuse of artemisinin-based combinations may result in malaria parasite resistance, negating the need to investigate the true cause of fever and, more crucially, glossing over life-threatening infections that might be deadly. Overall, the objective of delivering effective malaria case management, improving parasitological testing availability, and meeting national and global targets may be jeopardized. Overdiagnosis and overtreatment of malaria have been observed in Nigeria [42], Tanzania [43] and Ghana [42]. Malaria treatment should not be prescribed unless malaria is confirmed. This will help in limiting resistance of malaria infection towards the anti-malarial drugs. The knowledge about malaria prevention and the successful implementation of malaria control efforts will depend on understanding by all people involved [44].

From this study, it could be deduced that even though mothers seeking treatment from Kintampo Municipal Hospital have a high knowledge on malaria preventive practices, it is poorly reflected in their practice. The study conducted in a city of Yaoundé among urban dwellers on malaria prevention practice was not significantly different as the study affirmed the assertion that even though the urban dwellers have good knowledge on malaria prevention measures, few people apply the preventive measures [1].

The study findings indicate that the participants employed various strategies to protect themselves against malaria, including the use of insecticide-treated bed nets (ITNs), mosquito coils, mosquito sprays, and good nutrition. Insecticide-treated bed nets (ITNs) have been proven effective in reducing malaria illness, severe disease, and mortality, particularly among children under 5 years old [30, 45, 46]. As a result, pregnant women are encouraged to use ITNs for sleeping. Indoor Residual Spraying (IRS) and ITNs are recommended as highly effective preventive measures, reducing mosquito-human contact through insecticidal effects and physical barriers, respectively. According to a report by the WHO, ITN coverage increased in Africa from 29% to 2010 to approximately half of the population at risk of malaria between 2015 and 2017 [47].

Despite these interventions, malaria still incurs substantial costs, with approximately $12 million spent annually on diagnosis and treatment, causing an estimated economic loss of 1.3% in highly endemic countries [48]. The use of mosquito coils, although popular in malaria-endemic regions, is not recommended as a preventive measure against mosquitoes [49]. Mosquito coils contain insecticides that slowly vaporize when lit, aiming to provide protection against mosquitoes. However, the smoke emitted by mosquito coils can contribute to indoor air pollution and potentially lead to acute respiratory infections (ARIs) and other illnesses [50]. While mosquito coils are widely used in developing nations due to their affordability and accessibility, they can emit harmful emissions such as carbon monoxide, particulate matter, polycyclic aromatic hydrocarbons (PAHs), aldehydes, ketones, and various volatile organic chemicals (VOCs) [49, 51]. These emissions result from incomplete combustion, intentionally designed to delay the release of the pesticide. Exposure to these pollutants may have adverse health effects, with particulate matter linked to ARIs and VOCs and PAHs associated with cancer [51, 52]. To summarize, the study findings emphasize the importance of ITNs as an effective preventive measure against malaria, while cautioning against the use of mosquito coils due to potential health risks associated with indoor air pollution.

The findings of this study also corroborated with a health facility base study in Ethiopia to establish the prevalence of malaria and KAP towards malaria among febrile patients at Chagni health centre, which concluded that participants have high knowledge on the causes of malaria, good attitude towards malaria and good preventive practices [53]. Aside all that, there were some misconceptions about the knowledge, attitude and practices. Similarities in study findings could be due to the fact that both studies were conducted in a hospital setting with adult population. The study has some limitations; it is admitted that because KAP ratings are based on self-reported actions, this study is susceptible to recall and courtesy bias. These claimed actions may not necessarily correspond to actual practices, emphasizing the importance of additional observational studies to corroborate the reported behaviors. Furthermore, when interpreting the data, the well-known KAP-GAP, which refers to the gap between knowledge, attitudes, and behaviours, should be considered. Furthermore, the study acknowledges the role of the larger health system and social-cultural-economic variables on adherence, which can alter reported behaviours. These aspects should be considered when interpreting the findings and extrapolating them to other scenarios.

## Conclusion

The findings of the study pointed out that, majority of the mothers were knowledgeable about the causes, signs and symptoms and preventive measures of malaria in children under 5 years old. However, few misconceptions still exit on the part of some mothers. It was seen that; formal education does have influence on mother’s knowledge on malaria. There was statistically significant association between mothers’ demographic information such as level of education, occupation, religion, and their attitude towards malaria prevention. The study found that ITN, mosquito coils, and mosquito spray were the most effective malaria-prevention methods used by mothers of children under the age of five. Even though, mothers have good knowledge on the preventive methods of malaria, a lot of mothers are still adamant in using the protective methods. Mothers go to hospital earlier to seek treatment when they suspect their children are having malaria and that is a positive sign in the ongoing prevention of mortalities related to malaria in children under 5 years. Interventions should intensify sensitization targeted on the use of known preventive measures. Monitoring strategies to visit homes to encourage mothers on the use of preventive methods.

### Implication of the study

The study’s findings can assist improve public health strategies and programmes focused at reducing malaria prevalence and boosting preventative practices among mothers in rural areas. Policymakers and healthcare professionals can establish focused initiatives to address knowledge, attitudes, and practices gaps and promote effective malaria prevention measures by identifying gaps in information, attitudes, and practices. The study emphasizes the relevance of education in improving malaria preventive knowledge. The findings can be used to influence the development of educational campaigns aimed at boosting awareness about the causes, symptoms, and preventive measures of malaria among rural mothers. This can lead to more informed decision-making and more aggressive malaria prevention strategies. The study emphasizes the need of mothers seeking medical attention as soon as their infants show signs and symptoms of malaria. The findings can help politicians and healthcare practitioners emphasize the necessity of obtaining treatment as soon as possible and enhance rural access to healthcare facilities. This could include initiatives like expanding the availability of healthcare facilities, assuring treatment affordability, and raising knowledge about the consequences of postponing treatment. The research identifies shortcomings in the actual application of preventive measures among rural women. The findings of this study can help to improve the adoption and consistent use of preventative measures such as insecticide-treated bed nets (ITNs), adequate sanitation practices, and environmental management to reduce mosquito breeding places. The study can help lower the burden of malaria in rural regions and enhance the overall health outcomes of children under the age of five by addressing these gaps. The study gives unique insights into the malaria prevention knowledge, attitudes, and practices of women in a rural context. These findings can be used to guide future study on related themes, allowing researchers to investigate additional factors that influence preventive measures, assess the effectiveness of interventions, or investigate specific obstacles experienced in rural locations. This can contribute to a more comprehensive understanding of malaria prevention and support evidence-based public health decision-making. In summary, the findings of this study have the potential to inform public health interventions, improve educational campaigns, improve access to healthcare, strengthen preventive practices, and guide future research efforts in malaria prevention among mothers with children under the age of five in rural Ghana.

## Data Availability

Data can be obtained from the corresponding author on reasonable request.
